# Resilience mediates parenting style associated school bullying victimization in Chinese children and adolescents

**DOI:** 10.1186/s12889-022-14746-w

**Published:** 2022-12-02

**Authors:** Xue Chen, Jin Lu, Hailiang Ran, Yusan Che, Die Fang, Lin Chen, Junwei Peng, Sifan Wang, Xuemeng Liang, Hao Sun, Yuanyuan Xiao

**Affiliations:** 1 School of Public Health, Kunming Medical University, 1168 West Chunrong Road, Yuhua Street, Chenggong District, Kunming, 650500 Yunnan China; 2 Fuwai Cardiovascular Hospital of Yunnan Province, Kunming, Yunnan China; 3 The First Affiliated Hospital, Kunming Medical University, Kunming, Yunnan China

**Keywords:** Parenting style, School bullying victimization, Resilience, Children and adolescents

## Abstract

**Background:**

Existing evidence has shown that negative parenting style elevates the risk of school bullying victimization in children and adolescents. Resilience may play as a mediating factor in this association. However, this hypothesis has not been investigated.

**Methods:**

In this cross-sectional study, a total of 4582 Chinese children and adolescents had been surveyed by self-administered questionnaire. The Chinese version of Egna Minnen av. Barndoms Uppfostra (s-EMBU-C), the Olweus Bully/Victim Questionnaire (OBVQ) and the Resilience Scale for Chinese Adolescents (RSCA) were used to collect relevant information. Univariate and multiple logistic regression models were used to estimate the crude and adjusted associations between parenting style, resilience, and bullying victimization. Path analysis was used to estimate the mediation via resilience in the association between parenting style and bullying victimization.

**Results:**

After adjustment for possible covariates, the results of multivariate binary logistic regression model suggested that among all dimensions of parenting style, mother’s and father’s rejection were significantly associated with school bullying victimization. Path analysis revealed a statistically significant mediation of resilience in the association between parental rejection and bullying victimization, and among the five dimensions of resilience, emotion regulation, family support and interpersonal assistance accounted for the highest proportions of mediation.

**Conclusions:**

For children and adolescents who suffered from parental rejection, building up resilience, especially those measures aiming at improving emotion regulation ability and consolidating family and peer support, might be effective in reducing risk of school bullying victimization.

## Background

School bullying is prevalent worldwide. It is defined as physically violent behaviors, mockery, or verbal humiliations in a school setting, and can be perpetrated repeatedly over time [1]. In China, a previous review had estimated that up to 66% students had suffered from at least one type of school bullying [2]. School bullying affects both the well-being and social development of children and adolescents [3]. It is closely related to metal disorders like depression, anxiety, and stress [4]. Students can be involved in school bullying as bullies, victims, or both. Previously published studies revealed that victims of bullying were seen to have a higher risk of psychological problems and suicide attempts [5, 6].

It has been repeatedly supported that family environment plays a critical role in shaping the behaviors of children and adolescents. The positive association between parenting style and school bullying victimization has been well recognized. A previous meta-analysis of 70 studies disclosed that parenting style was significantly associated with bullying victimization [7]. In a more recently published cross-sectional study, researchers observed a prominent relationship between permissive parenting style and bullying victimization [8]. However, a direct intervention on parenting style related bullying victimization would be labor intensive and less effective, as most intervention programs aiming at cultivating positive parenting style yielded minimal impact [9]. With this regard, it is necessary to explore any factors that may be facilitating the relationship between parenting style and bullying victimization.

In the field of positive psychology, resilience (or psychological resilience) is the ability to cope mentally or emotionally with a crisis or return to pre-crisis status quickly [10]. It has attracted considerable study interest in the past years, especially among children and adolescents. In a recently published cross-sectional study, researchers found that perceived parenting styles were directly related to resilience in adolescents with addicted parents [11]. Another survey based on a national representative sample of 1204 American youth between the ages of 12 and 17 suggested that poorer psychological resilience was associated with higher risk of bullying victimization [12]. Therefore, it is reasonable to suspect that resilience may play as a mediator in the association between parenting style and bullying victimization. However, this assumption has not been specifically investigated. Once this suspected mediation by resilience really exists, it will provide a more realistic strategy to reduce the risk of parenting style related bullying victimization, given the fact that resilience can be substantially improved through short-term intervention [13].

Therefore, in the current study, we intended to preliminarily explore the mediation of resilience in the association between parenting style and school bullying victimization in a large sample of Chinese children and adolescents. We put forward the following two major hypotheses: 1) Resilience significantly mediates the association between parenting style and school bullying victimization; 2) The mediation of resilience varied for paternal and maternal parenting style.

## Methods

### Study design and participants

**Fig. 1:**
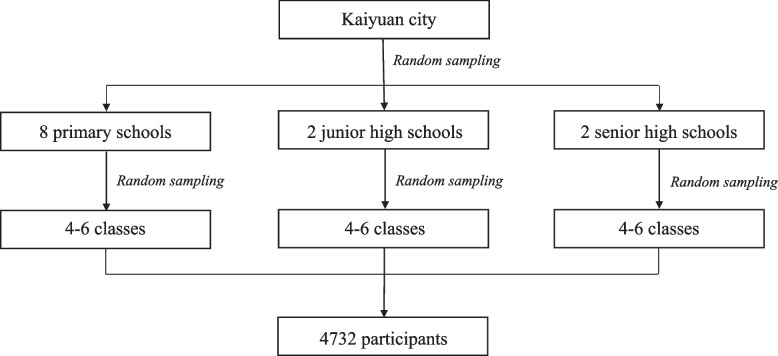
Sampling process of the current study

We conducted a cross-sectional survey in Kaiyuan, southwestern China Yunnan province from October 19 to November 3, 2020. Participants were selected using a two-stage simple random cluster sampling method. At first, 8 primary schools, 9 junior high schools, and 2 senior high schools were selected randomly. Then, in each chosen school, 4 to 6 classes were randomly selected, and all eligible students within the chosen classes were preliminarily included. The sampling process was depicted in Fig. 1.

The study population was determined as children or adolescents who aged 10-17. A minimum age of 10 was set as we simultaneously collected information on suicidal ideation and behaviors, and it has been suggested that only children aged above 10 can fully understand the concept and consequences of suicide [14]. Initially included subjects who satisfied either of the following conditions were further excluded: (1) Illiterate; (2) Physically ill, cannot finish the survey; (3) Auditory dysfunction or language disorder; (4) Unconscious or delirious, cannot clearly express oneself; 5) Refuse to participate. All participants were expected to finish a self-reported questionnaire composed of several modules measuring general characteristics, school bullying, resilience, and parenting style in sequential order. Prior to the survey, participants and their legal guardians were fully informed about the purpose of the current study, and there was no compensation for their participation. Written consents were obtained from legal guardians of the participants. The study protocol was reviewed and approved by the Ethics Committee of Kunming Medical University (No. KMMU2020MEC047).

### Measurements

#### Parenting style

The 21-item Chinese version of Egna Minnen av. Barndoms Uppfostra (s-EMBU-C) was used to assess the three dimensions (rejection, emotional warmth, over-protection) of parenting style. Rejection measures hostility, punishment, derogation, and blaming received from the parents (e.g. “My father/mother used to treat me in a way that embarrassed me”). Emotional warmth measures special attention, praise, unconditional love, support and affectionate from the parents (e.g. “I feel a warm, considerate and affectionate feeling with my father/mother”). Over-protection measures the extent of parents’ anxiety for the child’s safety, as well as intrusiveness (e.g. “My father/mother used to not allow me to do things that other kids can do, because he/she is afraid something will happen to me”). Paternal and maternal parenting style were measured separately for each question. The answer to each question was rated from “never” to “always”, with assigned scores from 1 to 4. The combined scores for the three dimensions are 6-24 for rejection, 7-28 for emotional warmth, and 8-32 for over-protection. The Chinese version of the questionnaire showed acceptable reliability and construct validity [15]. The Cronbach’s α for s-EMBU-C based on our analytical sample was 0.86 (Bootstrap 95% CI: 0.85-0.86).

#### School bullying victimization

We used the Chinese version of the Olweus Bully/Victim Questionnaire (OBVQ) to assess school bullying victimization [16]. The OBVQ has 2 parts with each contains 7 questions, measuring specific scenarios of bullying (e.g. “Hitting, kicking, pushing, shoving, or threatening”, “Calling mean nicknames or teasing”). All answers adopt a Likert 5-point scoring method: never happened, altogether once or twice, two or three times per month, once a week, and several times a week. Here in this study, bullying victimization was defined as “2-3 times per month” or more frequently been bullied, as recommended [17].

#### Resilience

The Resilience Scale for Chinese Adolescents (RSCA) designed by Hu and Gan was used [18]. The RSCA contains 27 items, which can be regrouped into 5 dimensions, namely goal concentration, emotion regulation, positive perception, family support, and interpersonal assistance (e.g. “Failure always makes me feel frustrated”, “When in need, I don’t know whom I can reach to”). All questions can be rated from 1 to 5 based on a 5-point Likert response, with a higher combined score reflects higher level of resilience. The Cronbach’s α for RSCA based on our analytical sample was 0.81 (Bootstrap 95% CI: 0.80-0.82).

### Statistical analysis

Descriptive statistics were used to describe and compare general characteristics of the participants. The direct associations between parenting style, resilience, and bullying victimization were explored by using univariate and multiple binary logistic regression models. Based on the results of multivariate models, the hypothetical path models were constructed to evaluate the mediation of resilience and its dimensions in the association between parenting style and bullying victimization.

All analyses were performed by using the R software (Version 4.1.1). Considering the unequal probability introduced by multi-stage simple random clustering sampling method, sampling weights were consistently adjusted for by using R analytical packages for survey data, such as “survey” and “lavaan.survey”. The significance level for statistical analyses was generally set as *p* < 0.05, two-tailed. However, for univariate logistic regression, a lower statistical significance of 0.10 was applied.

## Results

### General features of study subjects

A total of 4732 eligible adolescents were surveyed, 4582 provided valid and complete information, with an effective response rate of 96.8%. General features of the participants were displayed in Table 1: age ranged from 10 to 17, with a mean (standard deviation, SD) of 12.95 (2.0) years; a total of 665 respondents reported involved in school bullying, among which 573 were victims, with a bullying victimization prevalence of 12.5%; for two of the three dimensions of parenting style (emotional warmth and over-protection), mother’s parenting styles scores were higher (19 versus 18, 17 versus 16); the combined RSCA scores ranged from 40 to 131, with a median of 89 (Inter-quartiles range, IQR = 18).

**Table 1:** Characteristics of study participants, Kaiyuan, China, 2020 (*N* = 4582)

Characteristics	*N* (%)	Mean (SD)^§^/Median (IQR)^¶^
Gender		
Boys	2279 (49.7)	
Girls	2303 (50.3)	
Age (Years)		12.95 (2.0) ^§^
Ethnicity		
Han majority	1277 (27.9)	
Yi minority	1833 (40.0)	
Other minorities	1472 (32.1)	
Residence		
Township	1542 (33.7)	
Village	3040 (66.3)	
Grade		
Primary school	1588 (34.7)	
Junior high school	2440 (53.3)	
Senior high school	554 (12.1)	
Study style		
Day students	2294 (50.1)	
Boarding students	2288 (49.9)	
Father’s age (Years)		41.53 (6.0) ^§^
Mother’s age (Years)		38.96 (5.7) ^§^
Father’s education level		
Elementary school and below	1627 (35.5)	
Junior high school and above	2237 (48.8)	
Missing or unknown	718 (15.7)	
Mother’s education level		
Elementary school and below	1888 (41.2)	
Junior high school and above	1907 (41.6)	
Missing or unknown	240 (5.2)	
Marital status of the parents		
Married	3971 (86.7)	
Divorced	398 (8.7)	
Re-married or widowed	213 (4.6)	
Family income		
Stable	4127 (90.1)	
Unstable	455 (9.9)	
School bullying		
Uninvolved	3917 (85.5)	
Victim	573 (12.5)	
Bully	51 (1.1)	
Bully-victim	41 (0.9)	
Parenting style (s-EMBU-C sores)		
Father’s rejection		8 (3) ^¶^
Father’s emotional warmth		18 (8) ^¶^
Father’s over-protection		16 (5) ^¶^
Mother’s rejection		8 (3) ^¶^
Mother’s emotional warmth		19 (7) ^¶^
Mother’s over-protection		17 (5) ^¶^
Resilience (RSCA scores)		
Combined score		89 (18) ^¶^
Goal concentration		18 (5) ^¶^
Emotional regulation		20 (8) ^¶^
Positive perception		14 (5) ^¶^
Family support		20 (4) ^¶^
Interpersonal assistance		20 (6) ^¶^

*s-EMBU-C* Chinese version of Egna Minnen av. Barndoms Uppfostra Questionnaire

*RSCA* the Resilience Scale for Chinese Children and Adolescents

### Associations between parenting style, resilience, and bullying victimization

In this study, we only aim to investigate bullying victimization, therefore participants who were pure bullies (*N* = 51) or bully-victims (*N* = 41) were further deleted, 4490 participants reported “victim” or “uninvolved” were included into the analysis. Prominent demographic and socioeconomic covariates like sex, age, grade, study style, marital status of the parents, and income status were screened by univariate model at a lower significance level of 0.10.

A series of multiple binary logistic regression models were fitted subsequently. After adjusted for possible covariates, multivariate model 4 indicated that for all three dimensions of parenting style, only rejection was significantly associated with bullying victimization: every 5 points increase in father’s and mother’s rejection score were associated with an adjusted OR of 1.25 (95% CI: 1.05, 1.48) and 1.46 (95% CI: 1.11, 1.93), respectively. A 5-point increase in resilience score was related to statistically decreased odds of bullying victimization (OR = 0.91, 95% CI: 0.85, 0.97) (Table 2).

**Table 2:** Univariate and multivariate regression models for associated factors of school bullying victimization

Covariates	Univariate model	Multivariate model 1	Multivariate model 2	Multivariate model 3	Multivariate model 4
	OR (90% CI)	OR (95% CI)	OR (95% CI)	OR (95% CI)	OR (95% CI)
Age: + 1 year	0.81 (0.73, 0.90)	0.90 (0.79, 1.04)	0.89 (0.78, 1.03)	0.90 (0.77, 1.05)	0.91 (0.78, 1.06)
Sex (Ref: Boys): Girls	0.83 (0.70, 0.99)	0.90 (0.74, 1.09)	0.84 (0.68, 1.03)	0.86 (0.68, 1.09)	0.82 (0.65, 1.04)
Grade (Ref: Primary school)					
Junior high school or above	0.44 (0.30, 0.66)	0.61 (0.41, 0.90)	0.63 (0.42, 0.93)	0.60 (0.40, 0.91)	0.57 (0.37, 0.88)
Study style (Ref: Day students)					
Boarding students	0.63 (0.46, 0.86)	0.78 (0.59, 1.03)	0.78 (0.58, 1.05)	0.79 (0.57, 1.08)	0.79 (0.57, 1.10)
Marital status of the parents (Ref: Married)					
Divorced/Remarried/Widowed	1.73 (1.36, 2.20)	1.51 (1.17, 1.95)	1.53 (1.16, 2.01)	1.50 (1.13, 2.00)	1.48 (1.09, 2.01)
Income status (Ref: Stable): Unstable	1.41 (1.04, 1.91)	1.31 (0.97, 1.77)	1.31 (0.96, 1.78)	1.31 (0.94, 1.82)	1.30 (0.92, 1.83)
Father’s parenting style: + 5 points					
Rejection	2.01 (1.71, 2.36)	1.86 (1.59, 2.18)		1.28 (1.09, 1.52)	1.25 (1.05, 1.48)
Emotional warmth	0.75 (0.66, 0.85)	0.80 (0.69, 0.93)		0.78 (0.59, 1.03)	0.83 (0.61, 1.14)
Over-protection	1.18 (0.99, 1.40)				
Mother’s parenting style: + 5 points					
Rejection	2.07 (1.70, 2.51)		1.85 (1.48, 2.31)	1.56 (1.19, 2.04)	1.46 (1.11, 1.93)
Emotional warmth	0.79 (0.70, 0.88)		0.82 (0.73, 0.93)	1.02 (0.84, 1.24)	1.11 (0.96, 1.34)
Over-protection	1.27 (1.10, 1.48)		1.10 (0.93, 1.30)	1.08 (0.91, 1.28)	1.05 (0.87, 1.26)
Resilience: + 5 points	0.86 (0.82, 0.90)				0.91 (0.85, 0.97)

*RSCA* the Resilience Scale for Chinese Children and Adolescent

### Mediation of resilience

**Fig. 2:**
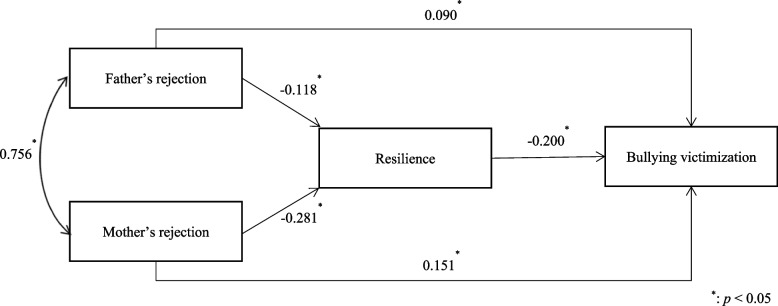
Path model illustrating mediation via resilience in the associations between father’s rejection, mother’s rejection, and bullying victimization

We put forward a hypothetical path model based on the previous analytical results to illustrate the possible associations between parents’ rejection, resilience, and school bullying victimization. As suggested by the analytical results, both father’s and mother’s rejection were indirectly associated with bullying victimization through resilience, the indirect associations via resilience accounted for 20.77 and 27.12% of the total associations between father’s and mother’s rejection and bullying victimization (Fig. 2).

**Fig. 3:**
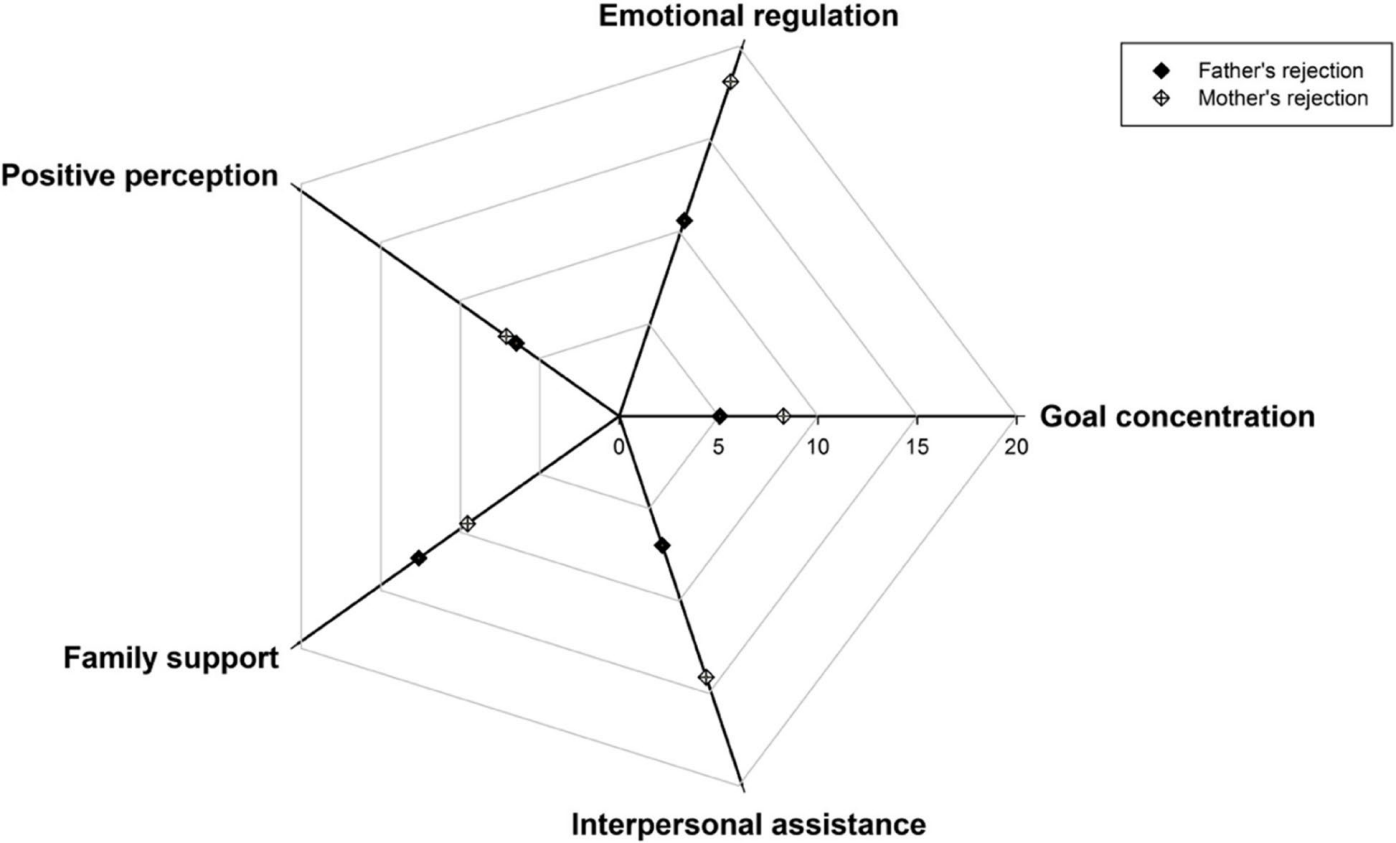
Percentage of mediation for different dimensions of resilience in the associations between parental rejection and bullying victimization

We further explored this statistically significant mediation of resilience by using its dimensions. The results suggested that the 5 dimensions (goal concentration, emotion regulation, positive perception, family support, and interpersonal assistance) of resilience played significant but discordant mediating roles in the associations between father’s rejection, mother’s rejection and bullying victimization: for father’s rejection, family support played the strongest mediation (12.46%), followed by emotion regulation (10.61%) and interpersonal assistance (7.02%); for mother’s rejection, emotion regulation played the strongest mediation (18.15%), followed by interpersonal assistance (14.17%) and family support (9.44%) (Fig. 3).

## Discussion

In the current cross-sectional study, we discussed the hypothesized mediation of resilience in the association between parenting style and school bullying victimization in a large representative sample of Chinese children and adolescents. We found that among the three dimensions of parenting style, only parental rejection was significantly associated with bullying victimization, and as expected, resilience significantly mediated this association. For mother’s rejection and father’s rejection, the 5 dimensions of resilience presented significant but discordant mediations, and among them, emotion regulation, family support and interpersonal assistance accounted for the highest proportions of mediation. All these important findings highlighted the promising role of resilience in preventing parenting style associated school bullying victimization among children and adolescents.

Rejection of parents may develop an indifferent environment in the family with high level of hostility, therefore children may evade expressing their needs or even adopt a submissive posture towards their parents in an effort to maintain their safety [19]. A newly published study has revealed an association between submissive behavior and cyber bulling victimization [20]. Moreover, brain alterations were found in children who had exposed to adverse parenting, they may present difficulties in learning independently and tend to be less assertive, which in turn make them more likely to be targets of bullying [21, 22]. Our study also found that mother’s rejection showed stronger association with bullying victimization than father’s rejection, the underlying causes for this phenomenon should be further investigated by future studies, especially through the culture perspective.

Path analysis suggested significant albeit discordant mediation via resilience in the associations between parental rejection and bullying victimization. Compared with father’s rejection, the mediation of resilience was stronger for mother’s rejection. Positive family environment helps children realize they can influence the situation and respond to when they perceived threats [23]. Although currently no pertinent studies on the mediation of resilience in parental style and bullying victimization have been published, a longitudinal study by Graziano et al. found that compared with fathers, mothers’ behaviors were more likely to predict reactive and effortful control capacity of children in response to frustration, an important underlying facet of “resilience” that we emphasized [24]. Our results suggested that for children and adolescents who are rejected by their parents, especially by their mothers, building up resilience may be effective for preventing risk of bullying victimization.

Further analysis revealed that, among the 5 dimensions of resilience, emotional regulation showed the strongest mediation in the association between mother’s rejection and bullying victimization. Previous studies have disclosed an important protective role of emotional regulation on reducing bullying victimization risk for children who grew up in negative family environments [23, 25, 26]. Prefrontal brain regions responsible for regulatory abilities continue to mature during adolescence, and these regions are especially susceptible to environmental influences, as a systematic review indicated [27]. Under this circumstance, victimization experience and emotional regulation process may influence each other bilaterally. Therefore, adolescence can be considered a critical stage for interventions to improve emotional competence, such as Mindfulness-Based Resilience Training (MBRT) [28], Penn resilience program (PRP) [29], and Integrative Body-Mind Training (IBMT) [30].

Among the rest dimensions of resilience, the prominent mediation by family support and interpersonal assistance cannot be ignored. It has been corroborated that family system plays a central role in the development of children’s social and emotional skills, which are relevant to their risk of bullying victimization. A study highlighted that family support may link parental stress and bullying involvement [31]. At the stage that children move from family to society, peer is the most important social connection outside of their families [21]. There is a consensus that children with overly intrusive or directive parents have a lower acceptance by peers and more negative peer interactions, and hence, are more likely to be victims of bullying [32]. Besides, a longitudinal study demonstrated that the effect of parental style on bullying forms was mediated by peer attachment relationships [33]. Another prospective study concluded that supportive relationships with peers and parents may play critical and complementary roles in protecting children against ongoing bullying victimization [34]. All these findings suggest that comprehensive prevention efforts focusing on improving interpersonal relationship can be considered to reduce the risk of parenting rejection related bullying victimization for children and adolescents.

The major findings of the current study stresses on the promising role of resilience based intervention measures in antagonizing parental rejection related school bullying victimization. Resilience is not hard to intervene for children and adolescents. Some intervention studies have already been done in Chinese adolescents and reached positive conclusions. For instance, adolescent peer education has been found effective in improving emotional regulation, family support, and interpersonal assistance [35], interpersonal assistance and emotional regulation had been significantly improved after mindfulness training [36]. However, the effectiveness of these proposed resilience intervention measures should be further validated by using randomized controlled trials of large representative samples.

Our study is among the first attempts to investigate mediation of resilience in the association between parenting style and bullying victimization in a large sample of Chinese children and adolescents. However, some limitations should not be ignored. At first, cross-sectional design of the current study prevents causal conclusions, the positive associations that we found should never be interpreted as effects. Besides, information bias will inevitably exist based on the self-reported questionnaires. Future longitudinal studies are warranted to further corroborate our major findings.

## Conclusion

In conclusion, our study identified a significant mediation of resilience in the relationship between parental rejection and bullying victimization. This finding probably suggests that, for children and adolescents who had experienced parental rejection, building up resilience, especially by using the measures that aiming at improving emotion regulation ability and consolidating family and peer support, could be effective in preventing risk of bullying victimization.

## Data Availability

The datasets used and analyzed during the current study are available from the corresponding author on reasonable request.
